# Data to assess the effect of moral identity on green consumption tendency

**DOI:** 10.1016/j.dib.2018.09.090

**Published:** 2018-10-02

**Authors:** Bo Wu, Zhiyong Yang

**Affiliations:** a Business School, Tianjin University of Finance & Economics, Tianjin, China; b Bryan School of Business and Economics, The University of North Carolina Greensboro, United States

## Abstract

This Data in Brief article is for Study 2 in the manuscript # JEVP-2018-140 (“The Impact of Moral Identity on Consumers’ Green Consumption Tendency: The Role of Perceived Responsibility for Environmental Damage”). It examines the effect of moral identity on green consumption tendency. The data was collected using online experiment. Participants were randomly assigned to moral-identity-activated condition and moral-identity-not-activated condition. The choice of eco-friendly product relative to conventional counterpart was measured. 162 Americans took part in the experiment. Data was analyzed employing SPSS. Logistic regression analysis was used as statistical tool of analysis.

**Specifications table**

**Table:** 

Subject area	Psychology
More specific subject area	Consumer behavior, green consumption, moral identity
Type of data	Table
How data was acquired	Experiment
Data format	Raw
Experimental factors	Sample consisted of online participants in United States
Experimental features	Moral identity is manipulated; Green consumption is measured through eco-friendly product choice relative to conventional counterpart
Data source location	United States
Data accessibility	Data is included in this article

**Value of the data**

• This data provides information on eco-friendly product choice relative to conventional counterpart across moral-identity-activated condition and moral-identity-not-activated condition using American sample.
• The effect of moral identity on green consumption can be compared with the effect of moral identity on other prosocial behavior.
• The effect of moral identity on green consumption can be compared with other cultures or countries.
• The data could be analyzed according to gender, age and whether the participants are born in the U.S.

## 1 Data

The data comprised experiment data on eco-friendly product choice relative to conventional counterpart across moral-identity-activated condition and moral-identity-not-activated condition, manipulation checks and demographics. Moral identity refers to a structured cognitive schema of moral values, goals, traits and behavioral scripts [1]. It was manipulated using the handwriting task. After writing their stories, participants completed a manipulation check for moral identity, using the question “*How much does the story you wrote in the handwriting task reflect how you see yourself as: (1) a member of an organization, (2) a moral person, and (3) safety conscious*?” (1 = *not at all*; 7 = *very much so*). Green consumption refers to the extent to which consumers consider the impact of their own behavior on the environment when they purchase, use, or dispose of products, and try to minimize the negative impact and maximize the positive impact on the environment [2]. Green consumption tendency was measured using eco-friendly backpack choice relative to conventional backpack. Participants were asked to make a choice between Backpacks A and B (0 = *Backpack A*; 1 = *Backpack B*). Backpack A was more stylish and durable, while Backpack B was more eco-friendly. As a manipulation check, participants further indicated the relative degree between these two backpacks along the following two dimensions: (1) being more eco-friendly, and (2) being more durable (1 = *definitely Backpack A*; 7 = *definitely Backpack B*). Finally, participants responded to demographic questions, including gender, age, and whether they were born in the U.S. (0 = *no*; 1 = *yes*).

Manipulation checks reveal that participants in the moral-identity-activated condition (*M* = 5.88) thought their stories were more reflective of themselves as moral people compared to those in the moral-identity-not-activated condition (*M* = 3.91; *t* (154) = 7.75, *p* < 0.001). However, the extent to which participants saw themselves as members of an organization, or safety conscious did not differ across these two conditions (*p*׳s > 0.29).

In addition, Backpack B was perceived to be more eco-friendly than Backpack A, as the average score (*M* = 6.56) was greater than the mid-point (*M* = 4.0; *t* (161) = 33.48, *p* < 0.001), whereas Backpack B was perceived to be less durable than Backpack A, since the average score along this dimension (*M* = 1.50) was smaller than the mid-point (*M* = 4.0; *t* (161) = − 31.29, *p* < 0.001).

A logistic regression analysis on the choice of Backpack B, using moral identity as the independent variable, and demographic variables as covariates, revealed a significant effect of moral identity (*b* = 0.87, Wald *χ*^2^ = 5.51, *p* = 0.019). Consistent with our expectations, 36.36% of those in the moral-identity-activated group chose Backpack B, compared with 21.18% in the moral-identity-not-activated group. Gender, age, and U.S.-born had no effect on the backpack choice (*p*׳s > 0.09).

## 2 Experimental design, materials and methods

The data presented a quantitative research based on an experiment design to assess the effect of moral identity on green consumption [3]. Experiment method was deemed suitable for data collection. One hundred and sixty-two people in the United States (105 females; *M* age = 37.99, SD = 12.24) were recruited through M-Turk to participate in this study in exchange for a small monetary compensation.

Following previous research [4], [5], in the moral-identity-activated condition, we told participants that the purpose of the task was to examine how different types of self-image affect the way people tell stories about themselves. They were provided with nine words about the traits that are commonly associated with being a moral person (e.g., caring, fair, and kind; [6]), and asked to write a story or a few stories about themselves using each word at least once. In the moral-identity-not-activated condition, participants were told that the purpose of the task was to examine how people relate to the objects in the surroundings when telling stories about themselves. They were provided with nine words about common objects (e.g., desk, car, and book), and asked to write a story or a few stories about themselves using each word at least once. After writing their stories, participants completed a manipulation check for moral identity, using the question “How much does the story you wrote in the handwriting task reflect how you see yourself as: (1) a member of an organization, (2) a moral person, and (3) safety conscious?” (1 = not at all; 7 = very much so).

After the handwriting task, participants completed an ostensibly unrelated survey of product choice. In this part, all participants were asked to imagine a scenario in which they were planning holiday travel and needed a backpack. There were two options for them to choose from: Backpack A was more stylish and durable, while Backpack B was more eco-friendly. Participants were asked to make a choice between Backpacks A and B (0 = *Backpack A*; 1 = *Backpack B*). As a manipulation check, participants further indicated the relative degree between these two backpacks along the following two dimensions: (1) being more eco-friendly, and (2) being more durable (1 = *definitely Backpack A*; 7 = *definitely Backpack B*). Finally, participants responded to demographic questions, including gender, age, and whether they were born in the U.S. (0 = *no*; 1 = *yes*). The data was coded and inputted in SPSS version 25. Data was analyzed through logistic regression analysis.
